# Therapeutic approaches for the treatment of genetic and acquired cardiovascular calcification

**DOI:** 10.3389/fcvm.2025.1636432

**Published:** 2025-10-07

**Authors:** Kevin O’Brien, Hervé Husson, Yves Sabbagh

**Affiliations:** Research and Development, Inozyme Pharma, Boston, MA, United States

**Keywords:** pyrophosphate (PPi), medial vascular calcification, bisphosphonates, enzyme replacement therapy (ERT), ENPP1, tissue non-specific alkaline phosphatase (TNAP) and phosphate

## Abstract

Vascular calcification, the deposition of calcium-phosphate crystals in the vasculature, occurs through a complex interplay between cellular processes and biochemical factors that are yet to be entirely defined. Vascular calcification results in stiffening of the arteries and ultimately cardiovascular complications. Deposition can occur either in the intima or media layers of a vasculature through discrete mechanisms and underlying pathologies. Medial calcification, the subject of this review, occurs in a specific set of pathologies including genetic disorders, diabetes, and chronic kidney disease. There are currently no approved therapies for prevention of medial vascular calcification leaving this an active area of unmet therapeutic need. One of the key molecule involved in preventing vascular calcification is pyrophosphate (PPi), long known as a potent inhibitor of mineralization. Many therapeutic avenues, both historical and current, have focused on increasing the plasma concentration of PPi. This can be accomplished by direct PPi supplementation or by use of bisphosphonates, acting as non-hydrolysable PPi analogs, though both approaches have limitations. Newer therapies utilize recombinant ENPP1, which generates PPi by hydrolysis of endogenous ATP, an approach which is currently being evaluated in clinical trials. Another approach to elevate plasma PPi concentration is by preventing enzymatic degradation of PPi through inhibition of alkaline phosphatase. Alternatively, chelation of either phosphate or calcium, the key constituent minerals of calcification, using phosphate binders represent other approaches as well as the use of magnesium and vitamin K supplementation. This review will first briefly discuss the pathophysiology of medial vascular calcification and describe the disease conditions involved before surveying the different therapeutic interventions evaluated to address the medial vascular calcification in the setting of genetic diseases as well as chronic diseases. We will present a bench to bedside view of development discussing therapeutic evidence in animal models, clinical trials and their relevance and applicability to clinical development.

## Background

### Vascular calcification

**Figure 1:**
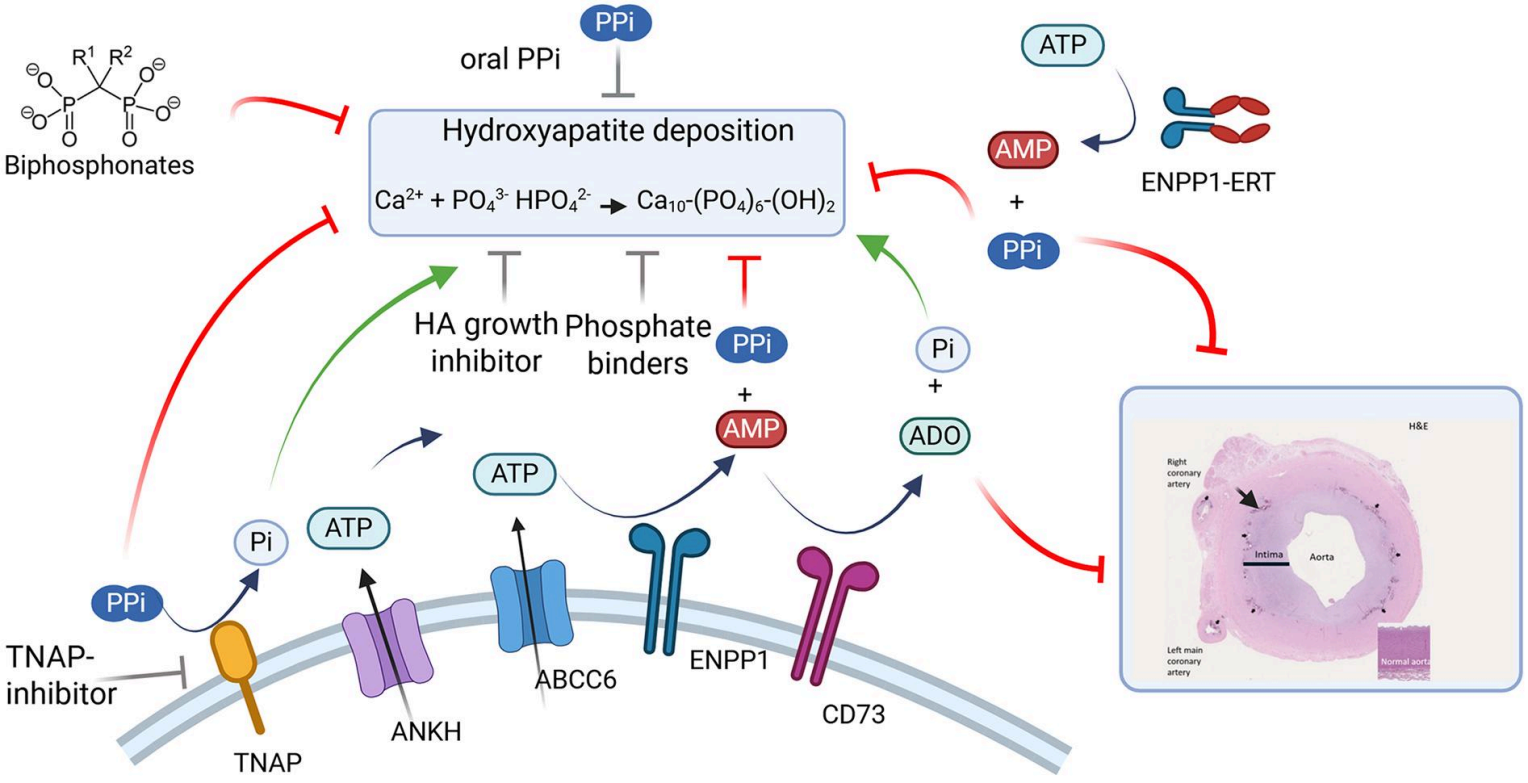
Pathways mediating medial vascular calcification and therapeutic interventions. Major enzymes and substrates modulating extracellular pyrophosphate levels. Black arrows represent enzymatic reactions and transport. Green arrows show molecules promoting vascular calcification. Red lines denote molecules that may prevent calcification (Bisphosphonates, TNAP-inhibitors (SB-425, DS-1211), hydroxyapatite growth inhibitors (SNF472), Phosphate binders (Magnesium carbonate, sevelamer), ENPP1 enzyme replacement therapy (INZ-701). Grey lines denote molecules with mixed or incomplete data for their roles in calcification. Inset image reproduced with permission from “Clinical presentation of ENPP1 deficiency” by Ferreira et al., licensed under CC BY 4.0. For illustration purposes, histopathology of the aorta of an ENPP1 deficient patient, showing pronounced thickening of the tunica intima (indicated by black line, both in affected aorta and in the insert depicting a normal aorta) with consequent luminal narrowing, as well as internal elastic lamina distorted by dystrophic calcification (black arrows). ATP, adenosine triphosphate; AMP, adenosine monophosphate; ADO, adenosine; PPi, pyrophosphate; Pi, inorganic phosphate.

Medial vascular calcification (MVC), the deposition of hydroxyapatite (HA) crystals in the medial vascular layer, results in altered hemodynamic parameters leading to neuropathy, peripheral limb ischemia, and increased cardiovascular mortality. MVC, which is caused by a variety of both genetic and acquired metabolic disorders, results in arterial stiffening due to the HA deposition. This in turn prevents normal hemodynamic regulation resulting in increased cardiac load which increases cardiac mortality (1). Recent advances in the pathophysiology of MVC have been made, however the exact mechanism leading to calcification remains elusive. In recent years, the imbalances in plasma metabolites, particularly phosphate (Pi) and pyrophosphate (PPi), as well as cellular signaling and resulting cellular transformation are thought to play a key role (2). In this review we aim to briefly review the role of the pyrophosphate pathway in MVC (Figure 1), survey disorders that are associated with MVC (Table 1), and then provide a review of the therapeutic options for MVC, including present therapies and those currently under clinical evaluation (Table 2).

**Table 1:** Corresponding genetic conditions with their respective genes involved in vascular calcification and OMIM numbers.

Gene (Abbreviation)	Condition	OMIM no
Tissue-nonspecific alkaline phosphatase (TNAP)	Hypophosphatasia (HPP)	241510 (infantile) 146300 (adult)
Inorganic pyrophosphate transport regulator (ANKH)	Calcium pyrophosphate deposition disease (Ankylosis)	118600
ATP binding cassette subfamily C member 6 (ABCC6)	Pseudoxanthoma elasticum (PXE), Generalized arterial calcification of infancy (GACI type 2), ABCC6 Deficiency	264800
Ectonucleotide pyrophosphatase/phosphodiesterase 1 (ENPP1)	Generalized arterial calcification of infancy (GACI type 1), Autosomal recessive hypophosphatemic rickets type 2 (ARHR2), ENPP1 Deficiency	208000
CD73	Arterial calcification due to deficiency of CD73 (ACDC)	211800

**Table 2:** List of clinical trials, conditions, therapies, and vascular/mineralization endpoints as discussed in the text.

Trial number or reference	Condition (ages)	Therapy (route of administration)	Clinical endpoints^a^
(48)	PXE/ABCC6 Deficiency (Adults)	PPi supplementation (Oral)	Plasma PPi
NCT04868578	PXE/ABCC6 Deficiency (>18 and <65 yrs)	PPi supplementation (oral)	Change in arterial calcification at 12 months compared to baseline as quantified by non-contrast CT, ABI, CIMT
(54)	PXE/ABCC6 Deficiency (≥18 yrs)	Etidronate (Oral)	Ectopic mineralization quantified with ^18^fluoride PET, carotid intima-media thickness, carotid-femoral pulse wave velocity, full body CT scan
NCT05832580	PXE/ABCC6 Deficiency (≥18 and <50 yrs)	Etidronate (oral)	Arterial calcification at 24 months as quantified by non-contrast CT, ABI, CIMT
NCT01585402	ACDC (≥18 and ≤80 yrs)	Etidronate (oral)	Ankle-brachial index, CT calcium score of lower extremities
NCT04686175	ENPP1 Deficiency (18 to <65 yrs)	INZ-701 (subQ)	Plasma PPi
NCT05030831	PXE/ABCC6 Deficiency (18 to <70 yrs)	INZ-701 (subQ)	Plasma PPi, CIMT
NCT06462547	ENPP1 and ABCC6 Deficiency (>1 yrs)	INZ-701 (subQ)	Plasma PPi
NCT05734196	Infants with ENPP1 and ABCC6 Deficiency (Birth to <1 yrs)	INZ-701 (subQ)	Left ventricular ejection fraction, low dose CT, plasma PPi
EUCT2024-512991-36-00	ENPP1 Deficiency (Birth to 17 yrs)	INZ-701 (subQ)	Plasma PPi, survival, Left ventricular ejection fraction, calcification of aorta and coronary arteries as measured by CT
NCT06046820	Pediatric patients with ENPP1 Deficiency (≥1 to <13 yrs)	INZ-701 (subQ)	Plasma PPi
NCT06283589	ESKD (>18 to <70 yrs)	INZ-701 (subQ)	Plasma PPi
NCT05569252	PXE/ABCC6 Deficiency (18 to 75 yrs)	DS-1211b (oral)	Plasma PPi, and Alkaline phosphatase levels
NCT01525875	PXE/ABCC6 Deficiency (≥18 yrs)	Magnesium (oral)	Von Kossa staining per unit area of dermis.
NCT02966028	ESKD (≥18 to ≤80 yrs)	SNF472 (IV)	Coronary artery calcification as measured by CT scan
NCT04195906	Calciphylaxis (≥18 yrs)	SNF472 (IV)	Absolute change in lesion size as measured by the Bates Jensen Wound Assessment Tool

^a^ Please refer to clinicaltrials.gov or cited publications for full list of clinical endpoints.

Listed endpoints highlight PD response related to vascular assessments.

yrs, years; CIMT, carotid intimal medial thickness; CT, computer tomography; ABI, ankle brachial index; ACDC, arterial calcification due to deficiency of CD73; subQ, subcutaneous; ESKD, end stage kidney disease; IV, intravenous.

### PPi homeostasis and role in vascular calcification

Pyrophosphate (PPi) has long been recognized as the key inhibitor of MVC (3). The role of PPi in inhibiting the formation of mineralization has been described since the 1960's (4). PPi functions by binding to, and inhibiting the growth of, HA crystals made of phosphate and calcium, as shown in both *in vivo* and *in vitro* studies (4, 5). The major enzyme responsible for extracellular PPi generation is ectonucleotide pyrophosphatase/phosphodiesterase 1 (ENPP1) (6). ENPP1 is a type II integral membrane protein consisting of two somatomedin B-like (SMB) domains, a phosphodiesterase domain, and a nuclease domain (7). ENPP1 is expressed in numerous tissues including liver, kidney, chondrocytes, and blood cells amongst others (8). ENPP1 can cleave a number of substrates, though two: adenosine triphosphate (ATP) and cyclic guanosine monophosphate-adenosine monophosphate (cGAMP), are known to play important biological roles, especially in the context of MVC (9). cGAMP is a key second messenger in the cyclic GMP-AMP synthase-stimulator of interferon genes (cGAS-STING) pathway, and ENPP1 is found upregulated on tumor cells presumably to degrade cGAMP to promote immune evasion, a role that has garnered immense interest in recent years (10). The best characterized role of ENPP1 is the catalytic cleavage of ATP into PPi and AMP. The latter is further broken down into adenosine and Pi by the actions of cluster of differentiation 73 (CD73) (11). PPi is cleaved into two Pi molecules by the action of tissue nonspecific alkaline phosphatase (TNAP) (12). Given that ATP serves as a substrate for PPi generation, the regulation of ATP export into the extracellular space is also critical for PPi homeostasis. The canonical modulator of ATP export is ATP-binding cassette subfamily C member 6 (ABCC6) (13). Another transporter, plasma membrane protein ankylosis homologue (ANKH), was long thought to transport PPi directly into the extracellular environment, though more recent studies have shown it transports ATP (14). Recently, PPi has also been implicated as having a role in gene expression, thereby contributing to inhibition of mineralization indirectly in addition to its more direct role in inhibiting the formation of HA crystals (15, 16). In this context PPi decreased the expression of *Enpp1* and *Ank* while increasing the expression of the mineralization inhibitor osteopontin. PPi has also been implicated in controlling expression of key osteoblast and osteocyte differentiation genes such as runt-related transcription factor 2 (*RUNX2*), bone gamma-carboxyglutamate protein (*Bglap*), and dentin matrix protein 1 (*Dmp1*) which in turn promote cellular differentiation and bone matrix deposition. These findings are based largely on experiments in cell culture so the role of PPi in gene expression and cell differentiation remains to be tested more robustly *in vivo*. Fibroblast growth factor 23 (FGF23), which is primarily expressed in osteocytes, plays a key role in phosphate homeostasis and plays an important role in MVC as well (17, 18). The mechanistic link between FGF23 and ENPP1 has not been defined precisely, but multiple publications show that FGF23 levels are increased in Enpp1 deficient mouse models and in ENPP1 Deficient patients (19–21).

## Diseases of abnormal calcification

MVC occurs in a specific subset of conditions, both genetic and acquired. ENPP1 Deficiency, is characterized by extensive medial vascular calcification and is caused by mutations in the *ENPP1* gene leading to a mortality rate of approximately 50% within the first 6 months of life. The majority of patients who survive the critical first year will develop autosomal recessive hypophosphatemic rickets 2 (ARHR2) which is characterized by hypophosphatemia, elevated FGF23 levels, joint pain, and rickets (21–23). ENPP1 deficient patients display very low plasma PPi levels which in turn leads to widespread ectopic calcification in the aorta as well as the renal, coronary, carotid, and iliac arteries (24). The heart and kidney are the most common locations of organ calcification in the disorder (22).

Another genetic disease that leads to low PPi is ABCC6 Deficiency, caused by variants in the *ABCC6* gene where the protein product is an important modulator of extracellular ATP levels. The infantile-onset phenotype of ABCC6 Deficiency, known as GACI Type 2 (GACI 2) has a similar clinical presentation to GACI 1 which is caused by mutations in ENPP1. The adolescent-adult phenotype of ABCC6 Deficiency, also known as pseudoxanthoma elasticum (PXE), is characterized by skin lesions, ectopic calcification, early calcification of Bruch's membrane in the eye, and progressive blindness (23, 25, 26).

Mutations in the *CD73* gene (also referred to as *NT5E*) cause the disorder arterial calcification due to CD73 deficiency (ACDC) which results in joint calcification and medial vascular calcification (27). Calcification occurs mainly in the lower limbs, leading to pain and limitations in mobility. CD73 is a membrane bound enzyme that metabolizes AMP to adenosine and inorganic phosphate (11). Adenosine is a potent inhibitor of intimal proliferation, which is a leading cause of stenosis that characterizes both ENPP1 and ABCC6 Deficiencies (28). While the link between CD73 and adenosine levels and thus stenosis is defined, the link between adenosine and vascular calcification is more convoluted. A variety of hypotheses exist to explain the link including adenosine control of ATP transport or expression of genes either directly implicated in the PPi/Pi pathway such as TNAP or genes involved in cellular differentiation (27, 29, 30). Additionally, AMP has been shown to inhibit ENPP1 activity, which in turn would lead to low PPi levels and an increased propensity for calcification (31). One of the key challenges in this field is the short half-life of adenosine in blood.

In addition to the genetic disorders described above, MVC is commonly observed in different stages of kidney disease, namely chronic kidney disease (CKD), end stage kidney disease, and calciphylaxis (1, 32). Chronic kidney disease impacts 11%–13% of the global population and is a major cause of mortality worldwide (33). CKD is graded into 5 stages based on glomerular filtration rate ranging from stage 1 representing a modest decrease in kidney function to stage 5 representing end-stage kidney disease (ESKD) (34). The leading cause of death for patients with CKD is not renal failure but cardiovascular disease (35). MVC is observed in CKD, particularly in the later stages of the disorder (36, 37). The reason for the appearance of MVC in CKD is complex and not entirely understood. Loss of kidney function corresponds with elevations in serum phosphate levels as the kidney is a key organ in phosphate homeostasis, where hyperphosphatemia itself is a risk factor for mineralization (38). Cellular trans-differentiation plays an important role, numerous reports have noted the development of osteoblast-like phenotype within vascular smooth muscle cells in the context of CKD (39). What drives this osteoblast phenotype is unclear with various reports implicating serum calcium, serum phosphate, chronic inflammation, and uremic toxins amongst others (39). CKD-mineral bone disorder (CKD-MBD) is the broad term used to define plasma mineral dysregulation, bone mineralization abnormalities, and ectopic calcification, specifically MVC, in the context of CKD (40). Despite the difference in etiology, there is a striking overlap between CKD-MBD and ENPP1 Deficiency, leading to the hypothesis that therapeutic solutions to the disorders could overlap. Closely related to the discussion of CKD-MBD is calciphylaxis, a disorder characterized by persistent necrotic skin lesions thought to be caused by calcification of arterioles (41). Calciphylaxis is most often associated with advanced stages of CKD and has a mortality rate of over 50% in the first year, although patients with no history of kidney disease are known (41). CKD, ESKD and calciphylaxis patients also have low PPi levels, supporting a common pathophysiology as a potential driver of MVC (41–44). There are currently no effective treatments for calciphylaxis (45).

## Potential therapeutic approaches

### Oral PPi supplementation

Given the central role of PPi in the pathogenesis of MVC, it is not surprising that the earliest therapeutic approach was simply to provide oral supplementation of PPi (46). Newer work has followed upon this approach showing that direct PPi supplementation can reduce MVC levels in both uremic and Abcc6^−/−^ pre-clinical models of MVC (47–49). While some success was seen in prevention of MVC in these models, the key challenge remains the short half-life of PPi. A subcutaneous injection of PPi had a measured half-life of around 30 min, limiting its potential as a viable therapeutic approach (49). Oral administration of PPi was also explored, with encapsulation protocols optimized to limit the unpleasant taste of PPi and poor gastrointestinal absorption (48). The impact of direct PPi supplementation has also been tested in an experimental rat model of CKD (50). In this model, the combination of adenine in feed plus calcitriol treatment caused kidney failure and aortic calcification. High concentration PPi supplementation was able to prevent aortic calcification. A concern with this approach is the degradation of the supplemented PPi into inorganic phosphate, which has the potential to exacerbate ectopic calcification. When healthy volunteers or PXE patients consumed PPi, their plasma phosphate levels increased with a single oral dose while PPi returned to baseline within a few hours of dosing (48). A new study investigating the impact of direct PPi supplementation in PXE involving a larger number of patients is currently ongoing (NCT04868578). Although PPi supplementation can prevent MVC in animal models, the low bioavailability and short half-life of PPi makes it challenging to envision as a viable therapeutic option.

### Bisphosphonates

Recognizing the short half-life of PPi, the natural next step in the therapeutic approach for MVC was to investigate the use of bisphosphonates to serve as nonhydrolyzable PPi analogs as inhibitors of MVC. Bisphosphonates have been in clinical use since the late 1960's for the treatment of osteoporosis, osteogenesis imperfecta, and other bone related diseases (51). Given the length of time that bisphosphonates have been in clinical use, multiple generations exist with differing mechanism of actions. The earliest bisphosphonates acted largely as non-hydrolysable PPi mimics consisting of two phosphates linked by a carbon atom and have a weaker inhibitory effect in the bone. Second and third generation bisphosphonates, consisting of phosphate groups linked to a central nitrogen atom act by inhibiting osteoclasts and are considered current front-line therapies for osteoporosis. Given the prominence of this class of drugs, numerous excellent reviews exist but we delineate the class distinction here to highlight that first generation bisphosphonates have better action on MVC and suggest that second and third generation bisphosphonates are not relevant as therapies for MVC given their primary mechanism of action on the bone (51, 52).

Studies using an *Abcc6*^−/−^ mouse model have shown that administration of etidronate, a first-generation bisphosphonate, reduced vibrissae calcification by about 50% (53). Interestingly, when the mice were treated with a second-generation bisphosphonate not only was ectopic calcification not prevented, but ectopic calcification increased. This highlights the importance of selecting the correct bisphosphonate as the mechanism of action on different tissues differs. A clinical trial consisting of 74 PXE patients divided between an etidronate group and a placebo group showed arterial calcification decreasing by 4% from baseline in the bisphosphonate group while increasing slightly in the placebo group (54). Another study specifically utilizing etidronate in the PXE population is currently ongoing (NCT05832580).

The use of bisphosphonates in Enpp1 deficient mouse models has been less conclusive. In one study, Enpp1 deficient mice were treated with a first-generation bisphosphonate with no reduction in MVC; however, bone parameters were improved (55). In a second study, Enpp1 mutant mice kept on a high phosphate diet were treated with bisphosphonates resulting in about 30% prevention of calcification in the skin and vasculature (56). Despite the lack of conclusive clinical evidence, the dearth of effective therapeutic options has led to the common use of bisphosphonates for patients with arterial calcification due to ENPP1 Deficiency. In a 2008 analysis, patients with GACI treated with bisphosphonates showed a higher survival rate (65%) compared to patients not receiving bisphosphonates (31%) (57). Another study found that 19 of 22 GACI patients treated with bisphosphonates survived infancy (58). While providing some evidence for the use of bisphosphonates in this population, both studies are hindered by small sample size and lack of true comparator groups for analysis. In a seminal publication consisting of a larger analysis of 247 patients and able to control for timing of bisphosphonate initiation, no statistical benefit on survival with the use of bisphosphonate was found in this patient population (23). Apart from the lack of evidence for efficacy, there are significant safety concerns with long-term bisphosphonate use, including reports of induction of rickets and osteosclerosis in pediatric patients receiving bisphosphonates (59, 60). Bisphosphonates also have a well described risk of osteonecrosis; these well documented side effects of bisphosphonates minimize their therapeutic potential, especially in a pediatric population that may be required to take these drugs for life (61).

The use of bisphosphonates in the context of ACDC has been the subject of a recent small clinical trial (NCT01585402) (62). While the trial only included seven patients, it was found that calcifications did not increase over the three-year treatment course. As this study did not include a placebo arm and therefore makes it difficult to understand if this represents a treatment effect, the lack of progression warrants further investigation.

Given the low PPi levels and bone fragility in CKD patients, the use of bisphosphonates has been explored both using animal models and clinically. In a surgical (5/6 nephrectomy) rat CKD model, first generation bisphosphonate administration resulted in reduced calcification in the aorta (63). Early clinical data, albeit with a small cohort size, showed that use of first generation bisphosphonates resulted in the prevention of aortic calcification in patients on dialysis (64). However, in more recent trials the results have been less compelling. In a retrospective study containing female patients with stage 3 or 4 CKD there was no impact of bisphosphonate on overall cardiovascular health (65). Another small study consisting of 46 CKD patients with stage 3 or 4 CKD failed to show an impact on vascular calcification in patients dosed with alendronate (66). Overall, there is conflicting clinical data on the efficacy of bisphosphonates in the context of CKD highlighting the need for larger and more controlled studies.

### ENPP1 enzyme replacement therapy (ERT)

As stated above, the major enzyme responsible for the generation of PPi is ENPP1. Therefore, an enzyme replacement therapy approach provides an alternate mechanism to increase PPi levels, whether due to ENPP1 Deficiency as well as deficiencies in other factors in the pathway such as ABCC6. In this approach, the catalytic domain of ENPP1 present in the extracellular domain is fused to an IgG Fc domain to create a soluble recombinant enzyme which can metabolize extracellular ATP as a substrate to generate PPi. Enpp1-Fc administration normalized the plasma PPi levels in Enpp1 deficient mice to WT levels and prevented ectopic calcification (67). In this early work the half-life of Enpp1-Fc was measured to be around 6.5 h, an improvement over the short half-life of direct PPi supplementation. Critically important was the improvement in survival in the mutant mice treated with Enpp1-Fc. All Enpp1-Fc dosed mice survived the entire length of the 55-day study while vehicle treated mice had a median lifespan of 35 days. Additionally, the bodyweight of the animals dosed with Enpp1-Fc was nearly identical to those of WT mice, compared to vehicle treated mice which were significantly lower. These results provided key data supporting the use of Enpp1-Fc as an enzyme replacement therapy.

INZ-701, a human ENPP1-Fc ERT developed for clinical use also showed the ability to correct plasma PPi, prevent mineralization, and restore bone microarchitecture in an Enpp1 deficient mouse model (68). The half-life of INZ-701 was close to 24 h in mice. INZ-701 was also tested in an Abcc6 deficient mouse model where it elevated plasma PPi levels and prevented mineralization, highlighting its potential to also be an approach for ABCC6 Deficiency, likely by hydrolyzing ATP potentially coming from ANKH modulation of ATP which might not be accessible to endogenous membrane bound ENPP1 but readily accessible to INZ-701 as a soluble ENPP1 which has a wide tissue distribution (69). In a rat model of CKD, MVC, and osteomalacia were induced through the use of an adenine diet and calcitriol injections. Dosing with INZ-701 resulted in the prevention of MVC and osteomalacia (70).

In addition to normalizing PPi levels the ERT approach recapitulates the entire biological function of ENPP1, breaking down ATP to form both PPi and AMP, allowing further processing of AMP by CD73 to form adenosine, a potent inhibitor of vascular smooth muscle cell (VSMC) proliferation. Pronounced vascular intimal proliferation and consequent luminal narrowing are observed in patients with GACI, contributing to arterial stenosis and cardiac complications (22). Notably, INZ-701 can prevent VSMC proliferation in an Enpp1 deficient mouse model through the restoration of adenosine levels (28, 71).

Given the promising preclinical data, INZ-701 is being evaluated in a variety of clinical trials. In a dose escalation trial of adults with ENPP1 Deficiency (NCT04686175), all doses of INZ-701 (0.2, 0.6, and 1.8 mg/kg twice weekly or 1.2 mg/kg once weekly) were able to elevate PPi levels into the healthy range which was sustained throughout the trial. The half-life of INZ-701 was calculated to be 126 h, supporting dosing once a week. Over the course of the study there was a dose-dependent decrease in plasma FGF23 levels and the bone turnover biomarker collagen c-telopeptide, as well as an increase in serum phosphate levels and bone specific alkaline phosphatase (72). In a study of ABCC6 Deficient adults (NCT05030831), INZ-701 increased PPi levels into the normal range in a dose-dependent manner, with the highest dose (1.8 mg/kg twice weekly) leading to consistent elevation of PPi into the normal range (73). The carotid intimal-medial thickness (CIMT), a measure of cardiovascular disease and implicated in stenosis could be due to low levels of adenosine which is a potent inhibitor of intimal proliferation, was stabilized or reduced upon dosing. This finding is particularly interesting as CIMT is measured noninvasively and is a known predictor of future cardiovascular complications (74). Additionally, retinal choroidal thickness, which has been shown to decrease with PXE progression, was increased in this population. Long term safety and efficacy amongst these patients are currently being evaluated (NCT06462547).

Given the favorable safety profile of INZ-701 and preliminary efficacy in the adult populations, INZ-701 is currently being evaluated for efficacy and safety in infants (NCT05734196 and EUCT2024-512991-36-00) and pediatric populations (NCT06046820 and EUCT2024-512991-36-00). Full results are forthcoming, but interim analysis in infants shows that 80% of infants receiving INZ-701 survived their first year compared to 50% in the natural history analysis. While data from pediatric patients is limited as the clinical trial is ongoing, dosed infants who survived to 1 year of age showed stabilized arterial calcifications, left ventricular ejection fraction, and lack of radiographic evidence of rickets. Additionally, INZ-701 has been evaluated in patients with end stage kidney disease with low PPi levels (NCT06283589). Treatment with 1.8 mg/kg weekly with INZ-701 elevated PPi levels to the healthy range within 3 weeks for all patients. These clinical studies provide proof of concept for the use of ENPP1-Fc for other diseases characterized by low PPi. While these preclinical and preliminary clinical results are promising, the data is limited to increases in plasma PPi into the normal range and more hard endpoints looking at vascular calcification are needed, which are being evaluated in ongoing clinical trials.

### Tissue non-specific alkaline phosphatase (TNAP) inhibitors

This review has up to now considered elevation of PPi via enzyme replacement therapy for the treatment of MVC. An alternative strategy to elevate PPi is to prevent its metabolism. One of the substrates of alkaline phosphatase is PPi, which is metabolized to generate two molecules of inorganic phosphate. A small molecule inhibitor of TNAP, termed SBI-425, was tested in a transgenic mouse model that overexpressed TNAP in the vasculature resulting in extensive vascular calcification, hypertension, and shortened lifespan (75). Inhibiting TNAP in this model leads to increased survival, 44 days in the vehicle group compared to 68 days in the SBI-425 treated group, with decreased aortic calcification upon dosing. The compound was then tested in an *Abcc6*^−/−^ mouse model (76). SBI-425 prevented mineralization in the vibrissae of this model. Interestingly, but perhaps not surprisingly, SBI-425 did not prevent mineralization in Enpp1 deficient mice (77). Elevation of PPi through the inhibition of TNAP relies on adequate existing PPi levels, which in an ENPP1 Deficient disease state are very low or even below the limit of detection. Thus, the inhibition of TNAP will not be effective in this background. TNAP inhibition was also tested in a mouse model of CKD that induced kidney damage and mineralization via a combination of adenine and high phosphate diets (78). In this context, treatment of mice with the inhibitor resulted in the prevention of mortality and decreased aortic calcification. Based on these data, a TNAP inhibitor, termed DS-1211, was developed for clinical use. In pre-clinical models (79, 80) DS-1211 has a half-life of just over 1 h in mice and is able to elevate the plasma PPi levels in *Abcc6*^−/−^ mice while decreasing the level of calcification of the vibrissae, confirming the results observed with SB-425. DS-1211was evaluated in a clinical trial of PXE patients (NCT05569252) for 12 weeks. Preliminary data is limited to highly variable, non-dose dependent increases in plasma PPi, and the short length of the trial will not be able to demonstrate effects on vascular calcification. In addition, there is a potential negative effect on bone with long term inhibition of alkaline phosphatase.

### Calcium and phosphate binders

Manipulation of the PPi pathway as the point of therapeutic intervention is a key approach for MVC in a variety of disease states. However, other approaches targeting either phosphate or calcium have been considered. Elevated phosphate levels predispose individuals to MVC (81). Magnesium carbonate, a compound that binds phosphate, thereby preventing mineralization, was added to mouse chow, and was able to prevent vibrissae mineralization in an *Abcc6*^−/−^ mouse model (82). These data have been more recently replicated using magnesium supplementation with very similar results (83). The ability of magnesium supplementation alone being able to prevent mineralization as well as the unchanging serum phosphate levels have suggested that the early results using magnesium carbonate may not actually be due to phosphate binding but may be the result of inhibiting vascular smooth muscle transformation preventing intimal proliferation (84). Magnesium supplementation prevents expression of *RunX2* and BMP-2, which has led to the hypothesis that magnesium functions by preventing the trans-differentiation of vascular smooth muscle cells (85). Direct magnesium supplementation has been evaluated in the PXE population with no significant impact on calcification levels (NCT01525875) (86).

Another phosphate binder that has been tested is Sevelamer, a polyallylamine polymer that binds phosphate in the intestine. A 40-person clinical trial of PXE patients split between a group receiving Sevelamer, a clinically approved phosphate binder for patients with CKD on dialysis, failed to significantly decrease vascular calcification (87). Phosphate binders have also been extensively clinically evaluated in the context of CKD. A recent meta review however, found that there was no evidence of systemic clinical improvement (88). Phytate, a well-known inhibitor of mineralization, acts by binding to calcium salts (89). SNF472, the clinical version of this compound, was tested for efficacy in a uremic model rat model and was able to reduce aortic calcification by around 70% compared to the vehicle treated group (90). SNF472 was also tested in an *Abcc6*^−/−^ mouse model resulting in a nearly 60% decrease in vibrissae calcification (91). In the same publication, rats were injected with FeCl_3_ to mimic the disorder calcinosis cutis. Treatment with SNF472 reduced skin calcification in this disease model as well. Based on these encouraging data, SNF472 was clinically evaluated in a phase 2b trial among patients with end stage kidney disease (NCT02966028) (92). Calcification was improved in some locations such as the aortic arch, but other areas of the vasculature were not impacted by treatment. A phase 3 study evaluating SNF472 in calciphylaxis patients was recently completed (NCT04195906) (93). No statistically significant benefit was noted upon dosing with SNF472 when compared to placebo in pain levels or wound healing, the two primary endpoints. Additionally, there was a pronounced, dose dependent, decrease in bone mineral density in individuals receiving the drug (94).

## Conclusions

Vascular calcification remains a worldwide health problem leading to high morbidity and mortality where currently no approved therapies exist. This review has covered different approaches that have been tested or are currently being evaluated in clinical trials to treat genetic and non-genetic disorders of vascular calcification. The strategies described involve small molecules, polymers, and enzyme replacement therapies. These approaches are either meant to increase the availability of inhibitors of calcification or prevent their metabolism. Although non-biologic agents have shown promise in animal models and early clinical studies, none have been successful in controlled clinical studies. Enzyme replacement therapies such as INZ-701 that address arterial calcification are advancing rapidly, particularly in ENPP1 Deficiency where INZ-701 provides the missing enzyme in this genetic disease. It is encouraging that clinical development is ongoing to evaluate these approaches in the clinical setting in the hope of finding a therapy to prevent or arrest the progression of vascular calcification. Additional research is critical for future generations of therapeutics targeting MVC. The underlying mechanisms that initiate and drive MVC are poorly understood, future therapeutic interventions, particularly those apart from the Pi/PPi pathway, require a much better understanding of the underlying cellular differentiation and role of adenosine as well as the identification of key biomarkers that can predict disease progression would also be of benefit to the field.
